# Orthoretroviral-like prototype foamy virus gag-pol expression is compatible with viral replication

**DOI:** 10.1186/1742-4690-8-66

**Published:** 2011-08-15

**Authors:** Anka Swiersy, Constanze Wiek, Juliane Reh, Hanswalter Zentgraf, Dirk Lindemann

**Affiliations:** 1 Institut für Virologie, Medizinische Fakultät "Carl Gustav Carus", Technische Universität Dresden, Dresden, Germany; 2 CRTD/DFG-Center for Regenerative Therapies Dresden - Cluster of Excellence, Biotechnology Center, Technische Universität Dresden, Dresden, Germany; 3 Angewandte Tumorvirologie, Deutsches Krebsforschungszentrum, Heidelberg, Germany

**Keywords:** Foamy virus, Gag-Pol fusion protein, retroviral morphogenesis, capsid assembly, Pol processing

## Abstract

**Background:**

Foamy viruses (FVs) unlike orthoretroviruses express Pol as a separate precursor protein and not as a Gag-Pol fusion protein. A unique packaging strategy, involving recognition of briding viral RNA by both Pol precursor and Gag as well as potential Gag-Pol protein interactions, ensures Pol particle encapsidation.

**Results:**

Several Prototype FV (PFV) Gag-Pol fusion protein constructs were generated to examine whether PFV replication is compatible with an orthoretroviral-like Pol expression. During their analysis, non-particle-associated secreted Pol precursor protein was discovered in extracellular wild type PFV particle preparations of different origin, copurifying in simple virion enrichment protocols. Different analysis methods suggest that extracellular wild type PFV particles contain predominantly mature p85^PR-RT ^and p40^IN ^Pol subunits. Characterization of various PFV Gag-Pol fusion constructs revealed that PFV Pol expression in an orthoretroviral manner is compatible with PFV replication as long as a proteolytic processing between Gag and Pol proteins is possible. PFV Gag-Pol translation by a HIV-1 like ribosomal frameshift signal resulted in production of replication-competent virions, although cell- and particle-associated Pol levels were reduced in comparison to wild type. In-frame fusion of PFV Gag and Pol ORFs led to increased cellular Pol levels, but particle incorporation was only marginally elevated. Unlike that reported for similar orthoretroviral constructs, a full-length in-frame PFV Gag-Pol fusion construct showed wildtype-like particle release and infectivity characteristics. In contrast, in-frame PFV Gag-Pol fusion with C-terminal Gag ORF truncations or non-removable Gag peptide addition to Pol displayed wildtype particle release, but reduced particle infectivity. PFV Gag-Pol precursor fusion proteins with inactivated protease were highly deficient in regular particle release, although coexpression of p71^Gag ^resulted in a significant copackaging of these proteins.

**Conclusions:**

Non-particle associated PFV Pol appears to be naturally released from infected cells by a yet unknown mechanism. The absence of particle-associated Pol precursor suggests its rapid processing upon particle incorporation. Analysis of different PFV Gag-Pol fusion constructs demonstrates that orthoretroviral-like Pol expression is compatible with FV replication in principal as long as fusion protein processing is possible. Furthermore, unlike orthoretroviruses, PFV particle release and infectivity tolerate larger differences in relative cellular Gag/Pol levels.

## Background

Spuma- or foamy viruses (FVs) are a special type of retroviruses that have adopted features in their replication strategy commonly found in both orthoretrovirinae and hepadnaviridae [reviewed in [1]]. In respect to their expression strategy for the overlapping viral capsid (Gag) and polymerase (Pol) open reading frames (ORFs), FVs do not follow the standard orthoretroviral transcription and translation mechanism, which includes Gag- and Gag-Pol fusion protein precursor expression from the same mRNA.

Orthoretroviruses express Pol exclusively as Gag-Pol fusion proteins from their full-length genomic RNA by ribosomal frameshift or termination read-through mechanisms [reviewed in [2]]. In human immunodeficiency virus (HIV), ribosomal frameshifting occurs at a frequency of 5-10% and involves two structural elements, a slippery heptamer at which the translating ribosome can slip by 1 nucleotide in the 5' direction, and a RNA secondary stem-loop structure as stimulator of ribosomal frameshifting 3' to the slippery sequence [3]. Retroviral ribosomal frameshifting or termination read-through not only permit Pol precursor synthesis, but also are essential for maintenance of the specific ratio of Gag-Pol to Gag precursor proteins. For orthoretroviruses an adequate ratio of these two precursor proteins is critical for capsid assembly, infectivity, and incorporation of the viral RNA genome [4-8]. It is generally believed that orthoretroviral Gag-Pol is incorporated into the virion via interactions with the Gag precursor, although particle association of Pol has been reported for murine leukemia virus (MLV) and HIV, when artificially expressed as a separate protein [9,10]. Orthoretroviral Gag-Pol copackaging is dependent on both the major homology region and adjacent C-terminal capsid sequences that are present in both proteins. The Gag-Pol precursor itself is unable to correctly assemble into infectious orthoretroviral particles.

FVs express Pol independently of Gag as a separate precursor protein that is translated from a singly spliced subgenomic mRNA [reviewed in [11]]. FVs seem to regulate the relative cellular expression levels of Gag and Pol by the use of a suboptimal Pol splice site [12]. As a consequence to this unusual Pol biosynthesis FVs have developed a special strategy to ensure Pol particle incorporation, essential for generation of infectious virions. Both Gag and Pol precursor proteins of FVs bind to full-length genomic viral transcripts [13,14]. Additionally protein-protein interactions between Gag and Pol seem to be involved in this assembly process as well [15]. Furthermore, only the PFV Pol precursor p127^Pol ^and not its mature processing products p85^PR-RT ^and p40^IN ^are incorporated into virions that preassemble their capsids intracellularly, close to the centrosome in a B/D type fashion [13,16].

PFV RNA genome and Pol precursor protein packaging into capsid structures requires at least two *cis*-acting sequences (CASI and CASII) [reviewed in [17]]. These elements comprise the 5' UTR of the FV RNA genome including a 5' part of the Gag ORF (CASI, nt 1-645) as well as discontinuous regions within a 2 kb fragment of the 3' part of the Pol ORF (CASII, nt 3869-5884). Within these two CAS elements, regions essential for RNA and/or Pol encapsidation as well as PR activity have been characterized [13,14,18].

Here, we examined whether PFV replication is compatible with an orthoretroviral-like Gag-Pol expression. Different artificial PFV Gag-Pol fusion constructs, including in-frame fusions and ribosomal frameshift mediated fusions, were generated. They were characterized in a proviral as well as in a replication-deficient vector system context to examine the effects of orthoretroviral-like PFV Gag-Pol fusion protein expression on virion morphogenesis, release, and infectivity. In particular, we were interested in determining whether, similar to orthoretroviruses, the ratio of FV Gag to Gag-Pol fusion proteins is very critical for particle morphogenesis. Furthermore, we determined whether unprocessed PFV Gag-Pol fusion proteins alone support capsid assembly and release.

## Results

### Release of non-particle associated PFV Pol protein

**Figure 1:**
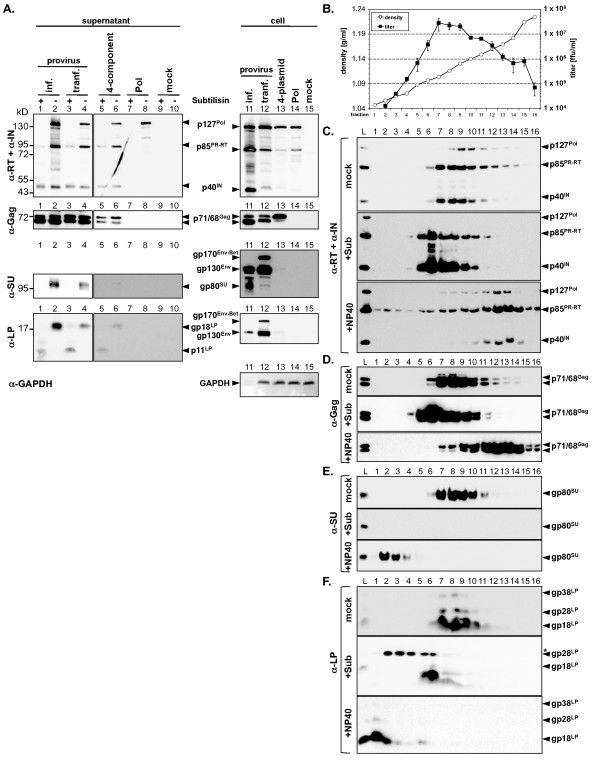
**Analysis of PFV Pol particle association in virus samples of different origin**. A) Western blot analysis of viral particle preparations of different origin, concentrated by ultracentrifugation through 20% sucrose and digested by subtilisin (+) or mock incubated (-) prior to lysis, using antibodies specific for PFV p85^PR-RT^and PFV p40^IN ^(α-RT + α-IN), PFV Gag (α-Gag), PFV Env LP (α-LP), PFV Env SU (α-SU), or mouse GAPDH (α-GAPDH) as indicated. Cell culture supernatants (30 ml total) were harvested after transient transfection of 293T cells (six 10 cm dishes per sample) with 16 μg wild type proviral expression construct pczHSRV2 wt (provirus transf., lane 3+4 [15 ml sup], lane 12 [1/30 10 cm dish]), transient co-transfection with 4-plasmids for a replication-deficient PFV vector system (4 μg puc2MD9, 4 μg p6iGag4, 4 μg p6iPol, 4 μg pczHFVenv EM002) (4-component, lane 5+6 [15 ml sup], lane 13 [1/30 10 cm dish]), transient transfection with the Pol expression construct p6iPol (4 μg + 12 μg pUC19) alone (Pol, lane 7+8 [15 ml sup], lane 14 [1/30 of a 10 cm dish]), or from infected BHK/LTR(HFV)lacZ cells (provirus inf., lane 1+2 [11 ml d9 MOI 1 infection sup], lane 11 [1/8 of a 175 cm^2 ^flask]). B-F) Linear velocity sedimentation gradient centrifugation analysis of PFV particles generated by transient transfection of 293T cells with the wild type proviral expression construct pczHSRV2 wt (forty-two 10 cm dishes, 210 ml supernatant total), concentrated by ultracentrifugation through 20% sucrose and prior pretreatment either by subtilisin digestion (+Sub, 60 ml supernatant equivalents), with 1% NP40 (+NP40, 90 ml supernatant equivalents), or mock incubated (mock, 60 ml supernatant equivalents). B) Infectious titer and density of the individual fractions from top to bottom (1-16). C-F) Western blot analysis of the load (lane 1, 1/12 of total) and the individual fractions F1-F16 (lane 2-17, 3/4 of total) using C) monoclonal antibodies specific for PFV Pol p85^PR-RT ^and p40^IN ^subunits (α-RT + α-IN), D) polyclonal antibodies specific for PFV Gag (α-Gag), E) monoclonal antibodies specific for PFV Env SU (α-SU), and F) polyclonal antibodies specific for PFV Env LP (α-LP). Subtilisin protein crossreacting with the PFV Env LP antiserum is marked with an asteriks. L: load.

During the course of this study we observed, in some control samples, the release of PFV Pol precursor protein p127^Pol ^into the cell culture supernatant when Pol was expressed alone after transient transfection of 293T cells (Figure 1A, lane 8). This apparently non-particle-associated Pol precursor protein was pelleted through 20% sucrose in a similar fashion as particle-associated Pol proteins and other viral structural proteins. A major difference was the absence of Pol cleavage products p85^PR-RT ^and p40^IN ^in supernatant pellets when Pol was expressed alone, whereas both processing products were present in the corresponding cell lysates (Figure 1A, lane 8, 14). In addition, this extracellular Pol precursor appeared to be present as free protein and not in a lipid membrane enveloped vesicular form because it was completely sensitive to subtilisin digestion (Figure 1A, lane 7, 8). This suggested that the PFV Pol precursor protein, but not its processing products, is released into the supernatant by non-conventional secretion mechanisms as it lacks a classical signal peptide sequence [19].

Pol precursor protein is frequently detected in PFV particle preparations of different origin [13,16,20,21]. To examine whether this really reflects not yet or incompletely processed particle-associated precursor protein or alternatively copurified extraparticular p127^Pol^, we generated wild type PFV particle preparations originating from various sources. Viral supernatants were obtained either by transient transfection of replication-deficient vector constructs and proviral expression vectors in 293T cells or alternatively from infected BHK/LTR(PFV)lacZ cultures. Subsequently, particles were concentrated by ultracentrifugation through 20% sucrose and duplicate samples were digested either with subtilisin or mock incubated. The analysis of the protein composition of these samples revealed that the majority of p127^Pol ^precursor present in these different particle preparations was sensitive to subtilisin digestion and therefore most probably was not particle-associated (Figure 1A, lane 1-6). In contrast, the Pol processing product p40^IN ^was resistant to subtilisin digestion whereas, in some experiments, a limited subtilisin sensitivity of the p85^PR-RT ^subunit was observed. PFV Gag p71^Gag ^and p68^Gag ^proteins were always insensitive to subtilisin digestion (Figure 1A, lane 1-6). Furthermore, as expected, the extracellular Env subunit gp80^SU ^was completely digested by subtilisin treatment whereas digestion of the LP protein gp18^LP ^removed only its extracellular C-terminal domain resulting in a protein with lower molecular weight (Figure 1A, lane 1-6).

To further support these observations, PFV particle preparations, concentrated by ultracentrifugation through 20% sucrose and pretreated with detergent, subtilisin or mock incubated, were separated by linear velocity gradient centrifugation on iodixanol gradients. Subsequently, the viral protein composition and infectivity of the individual gradient fractions were determined by Western blot analysis and titration on appropriate indicator cells, respectively. The result of such an analysis for replication-competent virus particle preparations generated by transient transfection of 293T cells with a PFV proviral expression construct is shown in Figure 1B-F.

Mock treated supernatants fraction 7 to 9, with densities of 1.10 to 1.13 g/ml, harbored the highest infectious virus loads (Figure 1B, fractions 7-9) and coincided with the strongest protein signals for Gag and Env (Figure 1D-F, fractions 7-9 upper panels). For Pol proteins the result was different. The highest amounts of Pol processing products p85^PR-RT ^and p40^IN ^were in accordance with the fraction infectivities (Figure 1C, upper panel). In contrast, a shift toward higher density fractions associated with lower infectivities was observed for the amount of p127^Pol ^precursor protein present in these particle preparations (Figure 1C, upper panel). Subtilisin digestion of concentrated particles prior to velocity gradient centrifugation led to a shift of the major PFV particle containing fractions (fractions 5-8) to a lower density and the complete removal of gp80^SU ^and p127^Pol ^but not the mature p85^PR-RT ^and p40^IN ^Pol subunits (Figure 1C-E, middle panels). The lower density of the subtilisin-digested PFV particles probably reflects the removal of the extracellular domains of the envelope subunits. NP40 treatment of virions prior and during velocity gradient centrifugation resulted in a shift of the major Gag and Pol protein containing fractions toward higher densities, probably representing membrane stripped PFV capsids (Figure 1C-D, lower panels). Furthermore, an overall broader density distribution of these proteins compared to untreated samples was observed, which might be an indication for an increased rate of disassembly of naked capsids (Figure 1C-D, lower panels). However, by this treatment no clear separation of Pol precursor and its cleavage products was observed (Figure 1C, lower panel). In contrast, Env subunits were physically separated from the Gag and Pol proteins, banding predominantly at very low densities (Figure 1C-F, lower panels). Interestingly, gp80^SU ^and gp18^LP ^proteins showed a different density distribution (Figure 1E+F, lower panels), which might suggest that they are found not in a detergent-resistant protein complex in the viral particle.

Taken together, these results suggest that particle-associated PFV Pol exists predominantly as mature p85^PR-RT ^and p40^IN ^subunits. Furthermore, Pol p127^Pol ^precursor protein, frequently observed in crude particle preparations, reflects mainly copurified extra-particlular Pol aggregates not enveloped by a lipid membrane. Therefore, a reliable statement on PFV Pol particle-association in crude virion preparations necessitates a subtilisin digestion prior to particle lysis and subsequent protein composition analysis as performed for the characterization of virions generated from Gag-Pol fusion protein mutants shown below.

### Cellular expression pattern of PFV Gag-Pol fusion proteins

**Figure 2:**
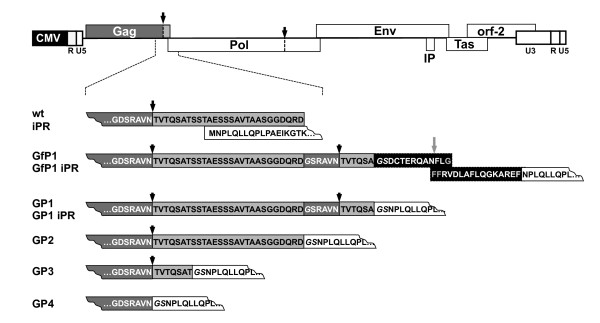
**Schematic illustration of PFV Gag-Pol expression constructs**. Schematic outline of the parental proviral expression construct pczHSRV2 wt. Below enlargement of the regions of Gag-Pol ORF overlap/fusion in the individual constructs as indicated. Sequences of PFV Gag origin in dark grey and light grey boxes, of PFV Pol origin in white boxes and of HIV-1 origin in black boxes. Amino acids (aa) are given in the 1-letter code. Aa not originally encoded by either PFV or HIV-1 derived sequences but from cloning sites are in italic. CMV, cytomegalovirus promoter; R, long terminal repeat region (LTR); U5, LTR unique 5' region; U3, LTR unique 3' region; IP, internal promoter; major PFV PR cleavage sites in PFV Gag and Pol are indicated by black arrows; HIV-1 PR cleavage sites are indicated by grey arrows.

PFV Pol naturally exists only as a separate Pol protein. To examine whether expression of an orthoretroviral-like Gag-Pol fusion protein is compatible with PFV replication, in particular virion morphogenesis, release and infectivity, we generated several constructs expressing artificial PFV Gag-Pol fusion proteins (Figure 2). We created expression constructs for pure in-frame Gag-Pol fusion proteins, differing only in their Gag domains and the presence of PFV PR cleavage sites between the Gag and Pol ORF (GP1, GP2, GP3 and GP4). In addition, we designed an expression construct separating PFV Gag and Pol ORF by a minimal HIV-1 Gag/Pol ribosomal frameshift site (GfP1). Translation of this construct's mRNA should result in a protein mixture, containing full-length PFV Gag with some additional HIV-1 Gag derived C-terminal amino acids (aa) and a PFV Gag-Pol fusion protein with an intervening PFV PR cleavage site and some HIV-1 Gag/Pol frameshift site encoded aa, at a ratio as observed for HIV Gag and Gag-Pol protein expression. For some constructs variants with catalytically inactive PFV PR (D_24_A mutation) were generated (GP1 iPR, GfP1 iPR) to examine the particle assembly and release potential of the Gag-Pol fusion proteins instead of a mixture of precursor protein and its cleavage products derived from the respective parental constructs.

**Figure 3:**
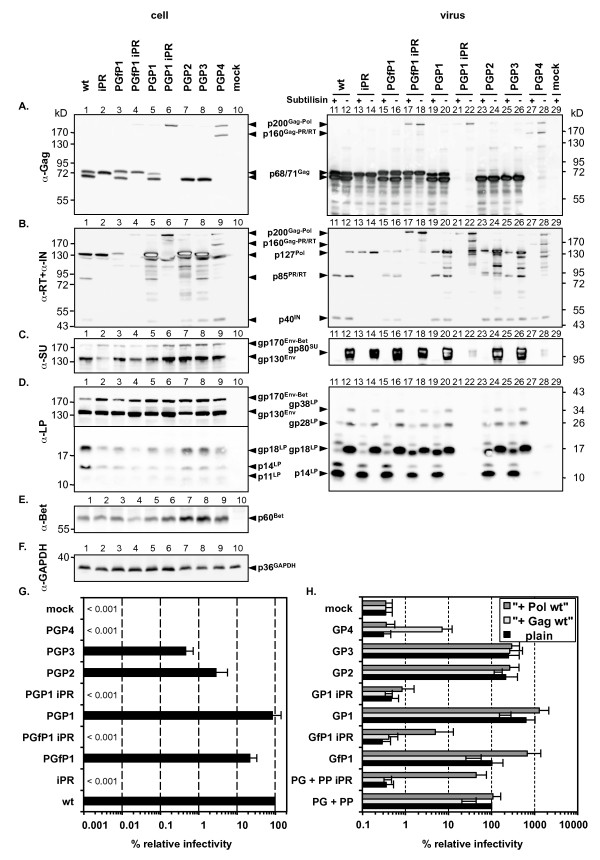
**Analysis of expression constructs for different Gag-Pol fusion proteins**. 293T cells were transiently transfected with the individual proviral expression constructs (A-E) or Gag/Pol packaging constructs in context of the replication-deficient 4-component PFV vector system (F) as indicated. Cell lysates (cell, lane 1-10, 1/25 of total) as well as viral particle preparations (virus, lane 11-29, 10 ml supernatant equivalents), concentrated by ultracentrifugation through 20% sucrose and either digested with subtilisin (+) or mock incubated (-) prior to lysis, were analyzed by Western blot. Antibodies or antisera used were specific for A) PFV Gag (α-Gag), B) PFV p85^PR-RT ^and PFV p40^IN ^(α-RT + α-IN), C) PFV Env SU (α-SU), D) PFV Env LP (α-LP) E) PFV Bet (α-Bet), F) rabbit GAPDH (α-GAPDH). The identity of the individual proteins is indicated in the middle, the molecular weight of the protein standard on both sides. 293T cells were transfected with: pczHSRV2 wt (lane 1, 11, 12; wt); pczHSRV2 iPR (lane 2, 13, 14; iPR); pczHSRV2 PGfP1 (lane 3, 15, 16; PGfP1); pczHSRV2 PGfP1 iPR (lane 4, 17, 18; PGfP1 iPR); pczHSRV2 PGP1 (lane 5, 19, 20; PGP1); pczHSRV2 PGP1 iPR (lane 6, 21, 22; PGP1 iPR); pczHSRV2 PGP2 (lane 7, 23, 24; PGP2); pczHSRV2 PGP3 (lane 8, 25, 26; PGP3); pczHSRV2 PGP4 (lane 9, 27, 28; PGP4); pcDNA3.1+zeo (lane 10, 29; mock). G) Relative titers of the individual 293T supernatants on BHK/LTR(PFV)lacZ cells. Means and standard deviations (n = 4) are shown. H) Relative infectivity of extracellular culture supernatants of 293T cells transfected with Gag/Pol packaging constructs in context of the replication-deficient 4-component PFV vector system using an eGFP marker gene transfer assay determined 3 days post infection. Means and standard deviations (n = 4-8) are shown. Identical amounts of Gag, Pol and Gag-Pol fusion protein packaging constructs were used. In case of the Gag-Pol fusion protein packaging constructs the total amount of transfected DNA was kept constant by the addition of empty pcDNA3.1zeo vector. Cotransfection of empty pcDNA3.1+zeo vector (plain), p6iGag4 (+Gag wt), or p6iPol (+ Pol wt).

First, we analyzed viral protein expression and infectious virus production after transient transfection of individual proviral expression constructs in 293T cells (Figure 3). The biochemical analysis of cell lysates demonstrated similar Gag, Env and Bet expression levels for the individual constructs (Figure 3A, C, D, E, lane 1-9). Some differences in the reactivity of the gp130^Env ^and gp170^Env-Bet ^precursor proteins to anti-SU and anti-LP antibodies were noted. The reason for this is currently unclear. However, preliminary data suggest that the anti-SU monoclonal antibody does not recognize all cell-associated PFV Env species equally well (data not shown). In general, only very low levels of Gag-Pol fusion precursor proteins were detectable (Figure 3A, B, lane 3-9). Only for the GP4 protein (PGP4), containing just the natural RT-IN cleavage site in the Pol coding sequences, as well as for the GfP1 and GP1 variants with catalytically inactive PFV PR domains (PGfP1 iPR, PGP1 iPR), higher fusion protein precursor levels were observed (Figure 3A, B, lane 4, 6, 9). In contrast, all fusion constructs with natural or artificial PFV PR cleavage sites at the C-terminus of the Gag coding sequences and active PFV PR domains (PGfP1, PGP1, PGP2 and PGP3) expressed Gag-specific products at levels comparable to the authentic proviral expression construct (Figure 3A, lane 1, 3, 5, 7, 8). Expression of p68^Gag ^was detectable in samples transfected with proviral constructs PGfP1, PGP1, PGP2 and PGP3, whereas it was absent in GfP1iPR and GP1iPR expressing cells (Figure 3A, lane 3-8). Gag precursor p71^Gag ^was only generated by expression constructs PGfP1 and PGP1 containing the full-length Gag ORF and C-terminal processing sites (Figure 3A, lane 3, 5).

In contrast, Pol expression of some constructs deviated significantly from wild type. The HIV-1 frameshift site containing construct PGfP1 and its PR-deficient variant PGfP1 iPR expressed lower amounts of Pol than the respective wild type counterparts (wt and iPR) (Figure 3B, lane 1-4). Quite the opposite was observed for most constructs having Gag and Pol ORFs fused in-frame (PGP1, PGP2, PGP3) that expressed higher amounts of Pol (Figure 3B, lane 1, 2, 5-9). Constructs with active PR domains and natural or artificial cleavage sites N-terminal of the Pol encoded sequences (PGfP1, PGP1, PGP2, PGP3) gave rise to p127^Pol ^precursor products (Figure 3B, lane 3, 5, 7, 8). For the GfP1 and GP2 Pol precursor proteins, the molecular weight was slightly increased in comparison to wild type (Figure 3B, lane 1, 3, 7). This most likely is due to the N-terminal presence of a HIV- Gag-Pol sequence or the PFV p3^Gag ^domain, respectively. Similar to wild type Pol these fusion proteins showed Pol precursor processing into p85^PR-RT^and p40^IN ^(Figure 3B, lane 7, 9, 11, 12). For the PGP4 construct, containing only the natural Pol PR-RT/IN cleavage site, p40^IN ^was observed at levels comparable to the other fusion proteins but no p85^PR-RT ^was detectable (Figure 3B, lane 9). Similar results were obtained using corresponding PFV Gag-Pol fusion protein packaging expression vectors of a 4-component PFV vector system, when transfected into 293T either alone or in combination with the residual vector system components (data not shown).

Taken together, this analysis revealed that all constructs expressed the predicted Gag-Pol fusion proteins, which were efficiently processed into the expected cleavage products. In comparison to wild type, relative cellular Pol expression was reduced when translation was controlled by a HIV-1 Gag/Pol frameshift site and increased upon in-frame fusion of PFV Gag and Pol ORFs.

### Particle release supported by PFV Gag-Pol fusion proteins

Analysis of the particle-associated protein composition revealed significant differences between the individual constructs (Figure 3, lane 11-28). First, a proviral expression construct having only the PR inactivated (iPR) displayed no particle-associated Gag and Pol processing (Figure 3A+B, lane 13, 14). However, unlike the wild type sample the p127^Pol ^protein of the iPR particle sample was only partially susceptible to subtilisin digestion (Figure 3B, lane 11, 13). This indicates that encapsidated, subtilisin-resistant Pol precursor is detectable if further particle-associated processing is prevented.

Second, the reduced cellular expression of Pol by the PGfP1 constructs resulted in somewhat lower amounts of particle-associated Pol protein (Figure 3B, lane 3, 15). However, unlike the wild type Pol precursor, the p127^Pol ^precursor protein present in PGfP1 samples was only partially sensitive to subtilisin digestion (Figure 3B, lane 11, 12, 15, 16). A similar phenomenon was observed for most of the constructs having Gag and Pol ORFs fused in-frame (PGP1 to 3), although the Pol precursor seemed to be more sensitive to subtilisin digestion than that of PGfP1 (Figure 3B, lane 15, 16, 19, 20, 23-26). This might point to a reduced PFV Pol processing capacity of these fusion proteins in comparison to wild type.

PGfP1 iPR, coexpressing p200^Gag-Pol ^and p71^Gag^, also showed only a partial subtilisin sensitivity of the p200^Gag-Pol ^precursor protein present in particle samples (Figure 3B, lane 17, 18). In contrast, the PGP1 iPR and PGP4 constructs, expressing only PFV Gag-Pol fusion proteins, contained hardly any subtilisin resistant particle-associated Gag-Pol fusion precursor proteins (Figure 3A+B, lane 17, 18, 21, 22, 27, 28). This indicates a strong deficiency in particle release of both constructs, which was further supported by the strongly reduced amounts of Env proteins detectable in these samples (Figure 3C+D, lane 21, 22, 27, 28). All other constructs showed capsid release similar to wild type (Figure 3A, lane 11-20, 23-26). Despite viral PR domain inactivation several Gag-Pol fusion protein cleavage products, but no mature p85^PR-RT ^or p40^IN ^subunits, were detected in the untreated PGP1 iPR particle samples using the anti-Pol antibody mixture (Figure 3B, lane 22). These cleavage products most probably represent partially degraded extraparticular Gag-Pol fusion protein since they completely disappear upon subtilisin pretreatment.

Taken together these results suggest that uncleaved Gag-Pol precursor proteins alone are unable to support efficient particle release, but can be incorporated to some extent in particulate structures if p71^Gag ^or p68^Gag ^is coexpressed.

### Infectivity of PFV Gag-Pol fusion protein mutants

Titration of viral supernatants generated by transient transfection of the different proviral expression constructs into 293T cells revealed a 5-, 30- and 200-fold reduced infectivity for the PGfP1, PGP2 and PGP3 constructs, respectively (Figure 3G). Only the PGP1 construct displayed wild type infectivity. The PGP4 and all constructs with inactivated PR (PGP1 iPR, PGfP1 iPR, iPR) were non-infectious. Thus PFV replication is compatible with orthoretroviral-like Gag-Pol expression strategies as long as processing between Gag and Pol domains is retained.

A similar picture was obtained when corresponding PFV Gag-Pol fusion constructs were examined in a replication-deficient 4-component PFV vector system, although overall infectivity of constructs with an active PR domain was somewhat increased in comparison to wild-type (Figure 3H, black bars), except GP4 that remained non-infectious. GfP1 derived supernatant showed wild type level of infectivity whereas those of GP2, GP3, and GP1 were 2-, 3-, and 7-fold increased. As expected all supernatant samples of constructs with inactive PR (PP iPR, GfP1 iPR, GP1 iPR) were non-infectious. Cotransfection of an identical amount of the wild type p71^Gag ^expression packaging construct led to a 2- to 5-fold decrease of infectivity in wild type (wt), GfP1, GP1 and GP2 samples. No change was observed for GP3 or PR inactive supernatants (PP iPR, GfP1 iPR, GP1 iPR) (Figure 3H, light grey bars). In contrast, the non-infectious GP4 construct was partially rescued by wild type Gag coexpression to levels of about 25% of the corresponding wild type sample (wt). Cotransfection of an identical amount of wild type p127^Pol ^expressing packaging construct resulted in no or only minor changes of infectivities in wild type (wt), GP1, GP2, and GP3 samples (Figure 3H, dark grey bars). The GfP1 sample was the only one where wild type p127^Pol ^coexpression led to a 7-fold increase in supernatant infectivity. Infectivity of non-infectious samples generated by cotransfection of separate wild type Gag and Pol iPR expression vectors (PG + PP iPR) could be rescued to 50% levels of the respective wild type control (PG + PP). In contrast, GfP1 iPR and GP1 iPR samples were complemented by wild type p127^Pol ^coexpression to a much lower extent if at all. This suggests that GP4 has a defect in Gag domains that can be partially rescued by wild type Gag. Furthermore, the Gag-Pol precursor fusion proteins with inactive PR domain seem to exert dominant-negative effects on wild type particle infectivity.

### Ultrastructural analysis of orthoretroviral-like Gag-Pol expression PFVs

**Figure 4:**
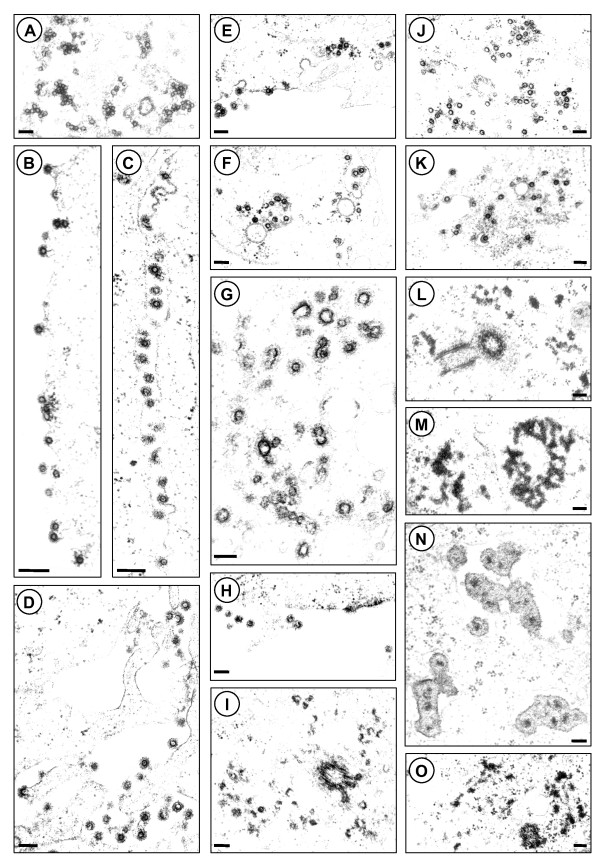
**Ultrastructural analysis of particle morphogenesis of proviruses expressing different Gag-Pol fusion proteins**. Electron micrographs showing representative thin sections of transiently transfected 239T cells using different pczHSRV2-derived proviral expression constructs as indicated. (A, B) wt; (C, D) iPR; (E, F) PGfP1; (G, H, I) PGfP1 iPR, (J, K) PGP1; (L, M) PGP1 iPR; (N, O) PGP4. Magnifications: (A) 15200×, (B) 31200×, (C) 29000×, (D) 19500×, (E) 14300×, (F, N) 15500×, (G) 23000×, (H) 15400×, (I) 15800×, (J, L, M) 14500×), (K) 13000×, (O) 13300×. Scale bar: 250 nm.

Subsequently, an ultrastructural analysis of 293T cells transfected with individual proviral expression constructs was carried out by electron microscopy to examine the effects of selected mutants on PFV particle morphogenesis. Representative electron micrographs are shown in Figure 4.

In cells transfected with the wild type proviral expression construct predominantly regular shaped capsid structures that were homogenous in size were observed as well as budding structures at plasma and intracellular membranes (Figure 4A, B). In contrast, more heterogeneous capsid structures were observed in samples transfected with the proviral expression construct having the PR inactivated (iPR) (Figure 4C, D). Many of these adopted a horse shoe-like morphology of apparently incompletely closed capsid structures, similar as reported previously for this type of mutation [22,23]. However, capsid structures with apparently normal morphology and budding structures at different cellular membranes were also detectable.

Cells expressing the PGfP1 proviral expression construct produced capsid structures and viral particles that closely resembled that of wild type (Figure 4E, F). In contrast, transfection of the PGfP1 iPR proviral construct, expressing p71^Gag ^and p200^Gag-Pol^, resulted in the appearance of very aberrant capsid structures at intracellular locations, but also membranous regions of the cell (Figure 4G, H, I). Their size and shape displayed a very broad heterogeneity and many incompletely closed capsid structures were observed. The morphogenic phenotype of the iPR mutation in the context of the ribosomal frameshift-mediated Gag-Pol fusion protein generated by PGfP1 (PGfP1 iPR mutant) was definitely more pronounced in comparison to the wild type translational scenario with Pol expressed as an independent protein (iPR mutant) (compare Figure 4C, D to Figure 4G, H, I).

Capsids and virions derived from the PGP1 expression construct were nearly identical to wild type (Figure 4J, K). However, if PGP1 was combined with the iPR mutation resulting in PGP1 iPR, which expresses only the p200^Gag-Pol ^protein, no structures with any similarity to original PFV capsids were detectable (Figure 4L, M). In contrast, electron-dense aggregated structures were observed at intracellular locations including the centrosome that might represent aggregated Gag-Pol fusion proteins. Similarly, in samples transfected with the non-infectious PGP4 proviral expression construct no regular PFV capsid structures or budding particles were detectable (Figure 4N, O). Here too, aberrant intracellular structures that might be aggregated PFV structure proteins were found. The electron-dense aggregated structures, most probably containing Gag-Pol fusion proteins, were only detected in these latter two samples and never observed in any other samples.

These results strongly suggest that PFV Gag-Pol fusion proteins alone are incapable of assembly into regular shaped capsid structures.

## Discussion

PFV polymerase biosynthesis and encapsidation are unique amongst retroviruses [reviewed in [1]]. First, Pol is expressed as a separate p127^Pol ^precursor protein [24-27]. Second, only the precursor but not the mature subunits p85^PR-RT^and p40^IN ^is encapsidated into the virion in a viral RNA genome-dependent manner that might involve additional Gag-Pol protein interactions [12,13,16,20,21,28].

During the characterization of PFV Gag/Pol constructs resulting in an orthoretroviral-like expression strategy in this study, we made an unexpected observation. In control particle lysates from cells transfected only with a PFV Pol expression construct the p127^Pol ^precursor protein was detectable, which was pelletable like intact PFV particles through 20% sucrose. However, this pelletable PFV p127^Pol ^seemed not to be enveloped by a lipid membrane, because it was sensitive to digestion by subtilisin, a non-membrane permeable endoprotease. Similarly, large amounts of predominantly subtilisin-sensitive p127^Pol ^precursor were detectable in partially purified PFV wild type virions from different sources, as reported previously [13,16,20,21]. In contrast, the mature p85^PR-RT^and p40^IN ^subunits in the same samples were mainly protected against subtilisin digestion. Furthermore, the amounts of Gag and Env processing products as well as p85^PR-RT^and p40^IN ^subunits, but not of p127^Pol ^precursor correlated with viral infectivity in fractions of linear velocity sedimentation gradient centrifugation runs that were used to further purify PFV particles. Together this strongly suggests that the majority of p127^Pol ^precursor detectable in the supernatant of PFV infected cells is not particle-associated.

The release mechanism of this non-particle-associated p127^Pol ^is currently unclear because it does not contain any classical signal peptide sequence potentially targeting it to the secretory pathway. Interestingly, only non-particle-associated p127^Pol ^was detectable in supernatant pellets from cells expressing PFV Pol alone, although intracellularly p85^PR-RT^and p40^IN ^cleavage products were observed. One explanation for this might be the failure of potentially co-released mature Pol subunits or mature Pol subunits derived from secreted p127^Pol ^precursor protein to be pelleted through 20% sucrose. Alternatively, indeed only the p127^Pol ^precursor is released, and its further processing is prevented. Potential reasons for such a processing defect could be a misfolding of the released p127^Pol ^precursor resulting in a lack of PFV Pol proteolytic activity mediated by oligomerization through the IN domain as recently proposed [29], a lack of essential cofactors (e.g. the recently described regulatory PARM viral genomic sequence element [18]), or a direct inhibition of PFV PR activity by factors of the extracellular environment. However, the observation that non-particle-associated, pelletable PFV Pol retains its RT activity [[16], and data not shown] at least seems to exclude a gross misfolding of the protein destroying all its enzymatic functions.

Expression of orthoretroviral Pol as a separate protein and not as the naturally translated Gag-Pol fusion protein was reported to result in assembly and release of infectious virions with particle associated Pol [9,10]. This indicates that orthoretroviral Pol can be packaged into capsids independent of the natural Gag-Pol fusion protein interaction with the Gag protein, although at reduced efficiency [9,10]. In contrast to our results, secreted pelletable Pol precursor or mature subunits were not observed upon orthoretroviral Pol expression as a separate protein [9,10]. Only Park *et al*. [7] described the release of HIV-1 Gag p24 and RT in the absence of detectable particle formation for cells transfected with proviral HIV-1 constructs expressing only in-frame Gag-Pol fusion protein and no separate Gag protein. Though, unlike non-particle associated PFV Pol, which can be pelleted by ultracentrifugation through sucrose, this was not possible for the HIV-1 Gag p24 and RT protein detectable in supernatants from cells transfected with this mutant proviral HIV-1 expression construct [7].

The observation of apparent extraparticle p127^Pol ^precursor protein detectable in pelleted PFV particle preparations is still in line with the current model of PFV Pol incorporation exclusively at its precursor state. However, it suggests that during or early after packaging efficient Pol precursor processing occurs, resulting in particle-associated PFV Pol predominantly as mature p85^PR-RT^and p40^IN ^subunits. If this is true then inhibition of further subunit processing after particle incorporation should result in the clear detection of particle-associated Pol precursor. Consistent with this is our observation of larger amounts of subtilisin-resistant p127^Pol ^precursor in crude virion preparations generated by constructs harboring a catalytically inactivated PFV PR domain (iPR).

The detection of large amounts of non-particle associated Pol precursor in crude PFV particle preparations of different origin represents another special feature of FVs. Furthermore, these data indicate that only mature PFV Pol p85^PR-RT^and p40^IN ^subunits are a good measure for particle-associated Pol in wild type PFV particle preparations. However, the results of the iPR mutants show that viral particles of mutants with a reduced or abolished proteolytic activity might harbor particle-associated Pol precursor, which can only be distinguished reliably from its non-particle associated counterpart by subtilisin digestion prior to particle lysis. This is an important aspect for the analysis of the different PFV Gag-Pol fusion construct of this study discussed below in greater detail, since the results from subtilisin analysis suggest that some of them display a reduced precursor processing capacity, which would have not been detected using standard virion protein composition analysis.

The characterization and analysis of expression constructs for different PFV Gag-Pol fusion proteins in this study revealed several interesting features of PFV. First, we demonstrate that PFV Pol expression in an orthoretroviral-like Gag-Pol fusion manner, even exclusively as in-frame Gag-Pol fusion, is compatible with PFV replication, which is true as long as proteolytic processing between Gag and Pol domains is possible. This is different to orthoretroviruses. Here in-frame Gag-Pol fusion expression in the absence of Gag coexpression is incompatible with viral replication [4,5,7,8]. In case of murine leukemia virus (MLV) and Rous sarcoma virus (RSV) in-frame Gag-Pol fusions abolish precursor processing, virion assembly and particle release [4,5]. On the contrary, spleen necrosis virus (SNV) and HIV-1 in-frame Gag-Pol fusion expression results in normal or enhanced structural protein expression associated with the failure to assemble and release infectious virions [7,8]. Thus PFV tolerates a much larger variation in the Gag to Pol ratio as orthoretroviruses do and processing by the viral PR is not abolished in a Gag-Pol fusion protein context. This might be a result of the unique PFV Pol packaging strategy with viral genomic RNA serving as a bridge between both components during capsid assembly, which might be a limiting factor determining the level of Pol incorporation. In line with this is the observation that all in-frame PFV Gag-Pol fusions, similar as reported for analogous orthoretroviral constructs [4,5,7,8], led to higher relative cellular Pol expression in comparison to authentic PFV Pol translation from a separately spliced RNA. However, the levels of particle-associated mature p85^PR-RT^and p40^IN ^of a full-length, cleavable in-frame PFV Gag-Pol expression (PGP1) were comparable to wild type and this correlated with infectivity. In contrast, ribosomal-frameshift mediated expression of PFV Gag-Pol resulted in lower cellular Pol levels compared to wild type. The infectivity of particles derived from HIV-1 frameshift site mediated Gag-Pol expression (GfP1) was reduced 5-fold and was reflected by reduced Pol particle incorporation and Gag precursor processing. Thus an optimal particle-associated PFV Gag to Pol ratio is important for maximal virion infectivity and might be regulated in part by interaction of PFV Pol with the viral RNA during capsid incorporation. Therefore increased cellular Pol precursor levels are not detrimental for PFV particle morphogenesis.

Second, there were significant differences of the various in-frame PFV Gag-Pol fusion constructs examined in the level of compatibility with viral replication. The in-frame GP2 and GP3 mutants displayed a similar Pol incorporation as GP1 but infectivity was reduced 30- and 160-fold respectively. This can be best explained by the failure of both mutants to generate p71^Gag ^in addition to p68^Gag^. In the case of GP2, the p3^Gag ^domain is fused to the N-terminus of Pol and cannot be removed due to the absence of a PFV PR cleavage site whereas the GP3 construct has the p3^Gag ^coding sequence removed. Therefore, both constructs lead only to p68^Gag ^expression after fusion protein processing. It has been demonstrated previously that PFV particles composed exclusively of p68^Gag ^display a 100-fold reduced infectivity although particle release and morphology are indistinguishable from wild type [30,31]. The infectivity levels of GP2 and GP3 are in a similar range as reported for PFV particles solely composed of p68^Gag^. Interestingly, a proviral construct similar to GP3 has been described previously by Löchelt *et al*. when elucidating the Pol biosynthesis mechanism [27]. Similar to our results they reported a 100-fold reduced infectivity of PFV Gag-Pol in frame fusion within the p3^Gag ^coding regions. However, although the authors observed Gag-Pol fusion protein processing, Pol subunit processing was severely impaired. This is different to the phenotype of GP3. The reason for this is currently unclear.

The phenotype of the in-frame GP4 fusion construct was special since it was the only one examined in the proviral and vector system context that did not allow production of infectious PFV particles. The only difference between GP4 and GP3 is the absence of a functional p68^Gag^/p3^Gag ^cleavage site between the fused Gag and Pol ORFs. Therefore GP4 only contains the natural Pol PR-RT/IN processing site. This leads to fusion protein processing into mature p68^Gag^-p85^PR-RT^and p40^IN ^subunits, indicating that also PFV PR is still active with a large N-terminal protein fusion. However, neither the GP4 precursor nor the mature subunits are able to support release of infectious PFV virions. This is demonstrated by the absence of significant amounts of subtilisin resistant structural proteins in respective particle preparations and the failure to detect normal capsid structures upon ultrastructural analysis of cells expressing GP4. Interestingly, coexpression of wild type Gag but not Pol resulted in a partial rescue of viral infectivity. In this setting the essential viral enzymatic functions are provided by the GP4 protein. Therefore, this suggests that coexpressed wild type Gag and GP4 can interact, probably through their Gag domains, and generate particles that retain some infectivity. Thus GP4 seems to have a defect in capsid assembly involving its Gag domains presumably due to C-terminal addition of the large p85^PR-RT^Pol domain.

Inactivation of the PFV Pol PR domain always resulted in the failure to produce infectious particles. However, it had different effects on particle formation and release depending on the context of the expression construct. PR inactivation in the PFV wild type (iPR) or GfP1 context (GfP iPR), where wild type p71^Gag ^is coexpressed, allowed formation and release of subtilisin resistant capsid structures that contained significant amounts of Pol precursor respectively GfP1 precursor fusion protein. Though the capsid morphology of both types of virions was aberrant, it was more pronounced for the GfP1 construct. In contrast, GP1 iPR failed to support capsid assembly and release of significant amounts of subtilisin resistant capsids. These results suggest that similar to reports for orthoretroviruses PFV Gag-Pol fusion proteins alone are unable to support correct capsid assembly and release [5,7,8]. However, like orthoretroviruses coexpression of Gag can rescue the particle release defect [8].

## Conclusions

Taken together our study describes for the first time the release of significant amounts of non-particle associated, pelletable PFV Pol protein, that appear to have no proteolytic, but polymerase, activity. However, the mechanism of Pol secretion remains unclear. Furthermore, we demonstrate that PFV Pol translation in an orthoretroviral-like manner by a lentiviral frameshift mechanism or solely as an in-frame Gag-Pol fusion protein is compatible with viral replication as long as processing between Gag and Pol domains is retained. Finally, PFV seems to tolerate much larger variations in the Gag to Pol ratio than orthoretroviruses, presumably as a result of the unique Pol capsid incorporation strategy with viral RNA serving as a bridging molecule between both proteins.

## Methods

### Cells

The human kidney cell line 293T [32], the human fibrosarcoma cell line HT1080 [33], and the PFV indicator cell line BHK/LTR(HFV)lacZ [34] were cultivated in Dulbecco's modified Eagle's medium (DMEM) supplemented with 10% heat-inactivated fetal calf serum and antibiotics. G418 was added at a final concentration of 1 mg/ml to BHK/LTR(HFV)lacZ cells.

### Expression constructs

A schematic outline of the constructs used in this study is shown in Figure 2. In the enlargement of the Gag-Pol ORF overlap/fusion of the individual constructs the amino-acid sequence is given. All constructs are based on authentic, non-codon-optimized PFV ORFs. The 4-component PFV vector system consisting of the PFV Gag expression vector pcziPG4 (PG), the PFV Pol expression vector pcziPol (PP), the PFV Env expression construct pczHFVenvEM002 (PE), and the enhanced green fluorescent protein (EGFP) expressing PFV transfer vector pMD9 (PV), has been described previously [28]. In this study a variant transfer vector puc2MD9 [35] and modified PFV Gag (p6iGag4) and PFV Pol (p6iPol2) expression constructs were used, all containing a pUC19 backbone instead of the original pcDNA3.1 zeo backbone. Expression vectors for PFV Gag-Pol fusion proteins all based on the p6iGag4 construct were generated by removing the start methionine in PFV Pol and fusing the PFV Pol-ORF in-frame to the PFV Gag-ORF at different positions resulting in constructs GP1, GfP1, GP2, GP3 and GP4 as illustrated in Figure 2. In addition, the authentic Pol translation initiation codon in the 3' part of the Gag ORF naturally overlapping with the PFV Pol ORF had been inactivated without changing the Gag protein sequence. Further modifications of the individual constructs are the insertion of a second p68/p3 proteolytic cleavage site between the PFV Gag- and Pol-ORF fusion in GP1 and GfP1. GfP1 harbors in addition a minimal HIV-1 Gag-Pol frameshift element containing an HIV-1 NC/p1 PR cleavage site [36]. In GP2, PFV Gag and Pol ORFs are fused in-frame without any additional sequences. GP3 has most of the PFV Gag p3 domain removed leaving the natural p68/p3 cleavage site intact whereas in GP4 the PFV Gag p3 domain was completely removed by fusing PFV Pol to p68^Gag ^coding sequences. For some experiments, expression vectors for Gag-Pol fusion proteins with a catalytically inactive PR (GP1iPR and GfP1iPR) containing a Pol D_24_A mutation [23,37] were used as indicated. The amino acid sequences in single letter code of the Gag-Pol fusion regions of the different mutants, starting with the non-altered aa position PFV Gag G_613 _and terminating with PFV Pol L_9_, are shown in Figure 2. Expression constructs for the Gag-Pol fusion proteins were generated by standard PCR cloning techniques and were verified by sequencing analysis. Further details on the cloning procedures and mutagenesis primers used are available upon request. The different PFV proviral Gag-Pol fusion constructs depicted were generated by replacing a 1829 bp *Swa*I/*Pac*I fragment of the infectious molecular clone pczHSVR2 [38] with the corresponding fragment of eukaryotic constructs expressing only the mutant Gag-Pol fusion proteins. Proviral constructs are labeled with the prefix "P" (e.g. PGP-1) that is omitted in Gag/Pol packaging constructs of the replication-deficient 4-component PFV vector system (e.g. GP-1).

### Transfections and particle concentration

PFV supernatants containing recombinant viral particles were generated as described earlier [39,40]. Briefly, replication-competent PFV supernatants were generated by transfection of proviral expression constructs into 293T cells as indicated using Polyethyleneimine (Aldrich) (16 μg total DNA per 10 cm dish) or Polyfect (Qiagen) transfection reagents (3 μg total DNA per well in 12-well plates). Replication-deficient PFV vector supernatants were produced by cotransfection of 293T cells with equal amounts of puc2MD9, pczHFVenvEM002, p6iPol and p6iGag4 or mutants thereof as indicated. Twenty-four hours posttransfection sodium butyrate (10 mM final concentration) was added to the growth medium for 8 h. Subsequently, the medium was replaced and harvested an additional 16-18 h later by passing it through a 0.45 μm syringe filter to obtain cell free culture supernatant (5 ml per 10 cm dish or 1.5 ml per well of 12-well plates) used for infection or particle purification. For particle concentration, extracellular viral particles were harvested by ultracentrifugation through a 20% sucrose cushion in SW28 rotors (Beckman) for 3 h at 25.000 rpm (112,000 g) and 4°C. After discarding the supernatant the particulate material was gently resuspended in phosphate-buffered saline (PBS) and further processed as indicated. For Western blot analysis an equal amount of 2× sodium dodecylsulfate protein sample buffer was added.

### Subtilisin treatment

Subtilisin treatment of concentrated particles was performed as described previously [41]. Briefly, for subtilisin treatment half of the particulate material resuspended in PBS was incubated for 2 h at 37°C in digestion mix containing final concentrations of 1 mM CaCl_2_, 50 mM Tris-HCl pH 8.0 and subtilisin (25 μg/ml). The other half was treated as a mock control by omitting subtilisin in the digestion mix. The digestion was stopped by adding Phenylmethylsulfonylfluorid (PMSF) at a final concentration of 100 μg/ml to the reaction mixture. For analysis of protein composition, an equal amount of 2× sodium dodecyl protein sample buffer was added to each reaction and the samples were separated by SDS gel electrophoresis and analyzed by Western blotting.

### Linear velocity sedimentation gradient centrifugation

After initial particle concentration by centrifugation through sucrose, described above, pelleted particulate material from forty-two 10 cm dishes (210 ml supernatant) resuspended in PBS, with or without prior subtilisin digestion, was carefully overlaid onto an 1.8 ml iodixanol (OptiPrep^®^) gradient in PBS containing of 200 μl layers ranging from 15% to 40%. In some experiments the gradient contained NP40 at a final concentration of 1% (v/v). Following ultracentrifugation at 48,000 rpm (197,000 g) and 4°C for 3 h (TLS55 rotor; Beckman) 120 μl fractions were collected from the top to the bottom. Subsequently, the infectivity of the fractions was determined by flow cytometry- or histochemistry-based assays, as well as the protein composition examined by Western blot analysis.

### Antisera, western blot expression analysis and quantification of particle release

Western blot expression analysis of cell- and particle-associated viral proteins was performed as described previously [37]. Polyclonal antisera specific for full-length PFV Gag [41], full-length PFV Bet or the leader peptide (LP) of PFV Env, aa 1-86 [37] were used. Furthermore, hybridoma supernatants specific for PFV reverse transcriptase (RT) (clone 15E10), PFV Integrase (IN) (clone 3E11), and the PFV Env surface subunit (SU) (clone P3E10) [42-44] were employed in some experiments. For loading controls commercially available polyclonal rabbit-anti mouse GAPDH (Sigma G9545) or monoclonal mouse-anti rabbit GAPDH antibodies (Sigma G8795) were used. After incubation with secondary anti-rabbit or anti-mouse antisera the blots were developed using Immobilion™ Western (Millipore) reagent. The chemiluminescence signal was digitally recorded using a LAS-3000 imager (Fujifilm) and analyzed using the Image Gauge software package (Fujifilm).

### Transductions

For analysis of particle preparations derived from proviral expression constructs BHK/LTR(PFV)lacZ cells were seeded 24 h prior to transduction with a density of 2 × 10^4^/ml in 12 well tissue plates and transduced with 1 ml of viral supernatant or dilutions thereof. After 48 h the amount of focus forming units (ffu) per ml was determined by a blue cell assay involving histochemical β-galactosidase staining [34]. All titration experiments were performed at least three times and the values in each independent experiment obtained with the wild type (wt) proviral expression construct pczHSRV2 were arbitrarily set to 100%. The titers of pczHSRV2 wt were in the range between 2-40 × 10^6 ^ffu/ml.

For analysis of replication-deficient FV vector-derived particle preparations, HT1080 cells were seeded 24 h prior to transduction at a density of 2 × 10^4^/ml in 12 well tissue plates and transduced with 1 ml of viral supernatant or dilutions thereof. The fraction of EGFP expressing cells was determined by flow cytometry analysis 72 h after transduction [28]. All transduction experiments were performed at least three times, and in each independent experiment the values obtained with wild type p6iGag4 and p6iPol2 constructs were arbitrarily set to 100%.

### Electron microscopy analysis

At 48 h post transfection, 293T cells were harvested and processed for electron microscopy analysis as described previously [45].

## Competing interests

The authors declare that they have no competing interests.

## Authors' contributions

CW and JR carried out the basic characterization of some of the constructs. HWZ performed the electron microscopy analysis. DL made substantial contributions to conception and experimental design of the study. Furthermore he was mainly involved in interpretation of data and drafting the manuscript. AS contributed to the experimental design, performed all main experiments on her own, coordinated and participated in collaborative experiments, and was involved in drafting the manuscript. All authors read and approved the final manuscript.
